# Evaluation of Cancer Cell Lines by Four-Point Probe Technique, by Impedance Measurements in Various Frequencies

**DOI:** 10.3390/bios11090345

**Published:** 2021-09-18

**Authors:** Georgia Paivana, Dimitris Barmpakos, Sophie Mavrikou, Alexandros Kallergis, Odysseus Tsakiridis, Grigoris Kaltsas, Spyridon Kintzios

**Affiliations:** 1 Laboratory of Cell Technology, Department of Biotechnology, School of Applied Biology and Biotechnology, Agricultural University of Athens, 11855 Athens, Greece; georpaiv@gmail.com (G.P.); skin@aua.gr (S.K.); 2 microSENSES Laboratory, Department of Electrical and Electronics Engineering, Faculty of Engineering, University of West Attica, 12244 Athens, Greece; d.barmpakos@inn.demokritos.gr (D.B.); mscres-38@uniwa.gr (A.K.); odytsak@uniwa.gr (O.T.); G.Kaltsas@uniwa.gr (G.K.)

**Keywords:** cancer cell lines, cell-based biosensor, doxorubicin, four-point probe measurements, polydimethylsiloxane

## Abstract

Cell-based biosensors appear to be an attractive tool for the rapid, simple, and cheap monitoring of chemotherapy effects at a very early stage. In this study, electrochemical measurements using a four-point probe method were evaluated for suspensions of four cancer cell lines of different tissue origins: SK–N–SH, HeLa, MCF-7 and MDA-MB-231, all for two different population densities: 50 K and 100 K cells/500 μL. The anticancer agent doxorubicin was applied for each cell type in order to investigate whether the proposed technique was able to determine specific differences in cell responses before and after drug treatment. The proposed methodology can offer valuable insight into the frequency-dependent bioelectrical responses of various cellular systems using a low frequency range and without necessitating lengthy cell culture treatment. The further development of this biosensor assembly with the integration of specially designed cell/electronic interfaces can lead to novel diagnostic biosensors and therapeutic bioelectronics.

## 1. Introduction

Cancer constitutes a complex disease mechanism as it involves interactions among cancer cells and their tissue microenvironments [1,2]. Doxorubicin (DOX) is considered the most common chemotherapeutic agent whose main mechanism of reaction affects the activation of apoptotic pathways, the intercalation into DNA, as well as the generation of reactive oxygen species [3,4,5]. Because of the many techniques used for the identification of the basis of the biological and physical properties of cancer cells require a long preparation time, highly trained personnel and expensive equipment [2] are critical to enhancing the basis for more direct prognosis of therapeutic effects using patient-derived tissues/cells for cell-based analysis.

An electrical impedance measurement provides a beneficial tool for the complex resistance in the presence of alternating current. This mainly depends on the frequency of the applied AC signal in conjunction with the biological tissue composition [6,7,8]. Thus, the frequency-dependent response of the biological tissues differs from the applied signal, taking into account the physiological and physiochemical conditions of the tissues. In addition, the value of the electrical impedance changes when responding to variations in health condition (e.g., disease, infection, etc.), composition, and biological tissue structure [9,10]. Through the study of the impedance of biological tissue (bioimpedance), valuable information can be obtained about both tissue physiology and anatomy, and hence the complex electrical impedance of the biological tissue appears to be a powerful tool used for non-invasive examinations of pathological or physiological status [11].

Multifrequency impedance analysis can provide valuable information considering the tissue’s features that contribute to the tissue’s characterization, since the response of the electrical impedance is frequency-dependent. Thus, plenty of research studies based on both single- and multi-frequency impedance analysis are very promising for noninvasively investigating the pathological or physiological health condition of the biological tissues.

The impedance measurement procedure can be implemented by using either two-electrode or four-electrode techniques. In order to perform an impedance measurement using two electrodes (two-point probe), both the current injection and the voltage measurement are implemented by the same electrodes. This method can cause considerable errors during the measurement procedure of the biological tissue, since the impedance of the electrode–tissue interface is not negligible and is added to the impedance of the measured sample, resulting in the perturbation of the final measured impedance [12,13]. In the three-point electrode configuration, the reference electrode is distinct from the counter electrode and it is connected to a third electrode, allowing for the measurement at a point close to the working electrode. This method is superior to the two-electrode setup as the potential alterations of the working electrode are measured separately from the alterations that might take place at the counter electrode [14]. The four-point probe method is applied in order to provide a solution to this problem. It consists of four electrodes placed in a straight line with an equal distance between them [15]. The two outer electrodes (current or driving electrodes) are used for the current injection of the sample, whereas the other two (voltage or sensing electrodes) are utilized for the determination of the resulting potential. This structure eliminates the effect of the electrode–tissue interface impedance during the measurements [12,13,15,16]. The increase in the distance among the electrodes for current and voltage can cause a decrease in the magnitude of the measured impedance [12,17]. The individual use of electrodes for the current injection and electric potential’s determination rules out the effect of the contact resistance between the electrodes and the sample. The two-electrode configuration system is restricted from the presence of electrode polarization impedance in the output signal, which is extracted at the same time as the measured signal [18,19]. On the contrary, in the four-electrode configuration, the polarization impedance is eliminated as the voltage electrodes do not carry current. Hence, the detection of the electrical properties of the biological tissue can be performed [20] while the effect of the contact impedance at the tissue, electrolyte, and the electrode interface is minimized [21].

Hence, in this study, we investigated the possibility of classifying different populations of various cancer cell line suspensions by their unique electrical impedance responses by utilizing a four-point probe method in order to rapidly assess the differences among them, as well as the instant interactions. The purpose of our investigation focused on monitoring both the phenotype alterations and behavior among the various cancer cell types and not on the development of a biosensor for the detection of a certain cancer biomarker. More specifically, four cancer cell types (SK-N-SH, HeLa, MCF-7, and MDA-MB-231) were cultured in population densities of 50 K and 100 K cells/500 μL. The effect of an anticancer compound (DOX) was also studied when applied to each cell type, so as to present differential cellular responses before and after drug treatment. A theoretical model was developed for the presentation of the main fitted features of the impedance responses for each cancer cell type in order to extract a unique signature for every cancer cell line. Our system could be further optimized as a non-invasive method for the identification of the cell status response when treated with an anticancer agent.

### Literature Review

In literature, a technique that utilizes the fundamentals of the electrochemical impedance spectroscopy can be also described as cell-based impedance. The characterization of intrinsic dielectric cell properties can be obtained using electrical impedance measurements. In the last decade, electrical impedance spectroscopy has become a possible method for the profiling of various cancer types in order to provide valuable information regarding capacitance, inductance, and impedance. The study of these electrical properties can be related to the behavior of cancer cell lines, in particular for monitoring their sensitivity and chemoresistivity [22].

By measuring the dielectric parameters of a cell culture and monitoring its metabolism and viability, information regarding cell proliferation can be obtained [23,24]. In the impedance technique, bioelectric recordings can be performed by utilizing either two-, three- or four-point probe configurations. Significant responses can be obtained with the use of the two-point probe system as both electrodes are utilized for the voltage measurement and the current injection [12]. The three-point probe setup dominates compared to the respective two-point probe setup as it allows for a measurement close to the working electrode due to the position of the reference electrode connected to the third one [14]. In the above-mentioned configurations, interactions occur between the electrodes and the tissues during the measurement procedure. The four-point probe methodology provides a solution to this problem as the two outer electrodes are used for the current injection and the remaining two for the voltage measurement, allowing for the monitoring of the electrical features in a biological tissue [20].

The application range of the cell-based impedance varies as this technique can contribute to the performance and non-destructive evaluation of the quality control of the cell cultures, providing significant and precise data. It can be applied for research in fields of toxicology and cancer, along with both 2D and 3D in vitro cell cultures [25]. Regarding cancer, many researchers have used electrical impedance measurements in order to analyze various cancer cell lines. More specifically, Wang et al. [26], reported an impedance sensor for the performance of a real-time cell viability analysis and the examination of the anticancer activity of three various drugs applied to a 3D in vitro model of human hepatoma cells (HepG2). A multidimensional impedance platform was introduced by Seidel et al. [27]. In order to test the anticancer activity in a single and combination treatment, they extracted a tissue from a patient for the development of a 2D and 3D cell culture model for melanoma cancer.

In a previous study, Bragos et al. [28] performed a rapid model for cancer cell growing, utilizing two-electrode and four-electrode interfaces in cell suspensions (CD34- cell line). As it was observed, at very low frequency range (<10 Hz) the two-electrode configuration interactions led to a difficulty regarding the classification of the cell types (in terms of size and morphology), whereas these obstacles were eliminated with the use of the four-electrode system [29].

In addition, Fing et al. [30] developed a microfluidic system for the differentiation of three various cancer cell lines, including HeLa, A549, and HepG2. This system combined both electrochemical impedance and impedance flow cytometry showing that, at higher frequencies, electrical properties prevail over not only the total impedance but also the cell size, leading to the successful differentiation of the cell lines [30]. Regarding cell spreading, adhesion, and proliferation, Yang et al. [31] presented the importance of these biomarkers for the discernment of cancer and non-cancer cell cultures. In particular, in their work, they analyzed the alterations between oral cancer cells (CAL 27) and non-cancer oral epithelial cells (Het-1A) using electrochemical impedance measurements [22].

## 2. Materials and Methods

### 2.1. Human Cell Culture and Application of the Anticancer Agent Solutions

Human neuroblastoma cell line SK-N-SH (ATCC^®^ HTB-11™), HeLa cervical cancer cells (ATCC^®^ CRM-CCL-2™), triple positive breast cell line MCF-7 (ATCC^®^ HTB-22™), and triple negative MDA-MB-231 (ATCC^®^ HTB-26™) were cultured in an incubator at 37 °C, 5% CO_2_ as described previously [32]. Doxorubicin hydrochloride (>98.0%) (DOX) (Fluka Chemie GmbH, Buchs, Switzerland) was initially dissolved in dimethylsulfoxide (DMSO). DOX was inserted into the cell suspensions’ medium in a final concentration of 1 mM before measurement. DOX concentrations used were particularly high in order to present their cytotoxic effects on cancer cells in a short time [33].

### 2.2. Trypan Blue Dye Exclusion Cell Viability Testing

The determination of cell viability in the cell suspensions was performed by the Trypan Blue dye exclusion assay [34] based on the principle that intact membranes of living cells exclude certain dyes, whereas dead cells do not. Equal parts of 0.4% trypan blue dye solution (Merck KGaA, Darmstadt, Germany) were mixed with the cell suspension, incubated for 2–3 min at 25 °C, and afterward inserted into the hemacytometer counter. All cells (clear and blue) in each large square in each of the four hemacytometer’s corners were counted by an inverted light microscope (ZEISS Axio Vert.A1, Carl Zeiss Microscopy, LLC, White Plains, NY, USA), and the pictures were processed by ZEN lite software. Each experiment was repeated four times. The percentage of viable cells was calculated by the following Equation:

%” Viable” = (“Nv”⁄NT) × 100,(1)

where Nv is the number of viable cells and NT is the number of total cells counted (living and dead).

### 2.3. Background-Experimental Setup

A complex electrical impedance can be described as the overall response of the biological tissues when an alternating electrical signal is applied. The electrical impedance is a quantity that depends on various parameters such as the signal frequency, tissue composition, tissue health, and tissue structure. For that reason, the impedance differs not only from tissue to tissue but also for measurements within the same tissue sample at different electrode locations. Moreover, the impedance of the biological tissue might have different values compared to other parts of the same tissue, considering the alterations in tissue health, physiology and pathology, morphology, and composition.

The electrical impedance of the tissue was measured by applying a sinusoidal alternating current with an amplitude in the order of 10 μA at different low frequencies (5 to 1000 Hz) through the electrodes attached to the tissue surface [15].

The four-point impedance methodology required four probes in the line. The current was applied in the two outer electrodes, thus the impedance was extracted by measuring the voltage in the two inner electrodes. The four-point probe impedance “Z” could be defined as the ratio V/I, where “V” is the measured voltage drop and “I” is the current passing through the sample [35].

**Figure 1:**
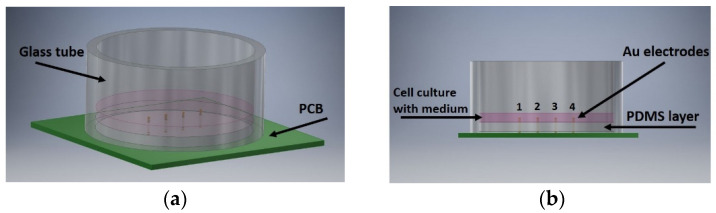
Experimental setup: (**a**) The glass tube is placed on the PCB including the four-electrode configuration. The PDMS layer is placed on the bottom of the tube, covering half of the electrodes, allowing the addition of the cell culture on the top; (**b**) cross-section of the experimental setup.

The experimental setup consisted of a cylindrical glass tube (d = 15.5 mm) that was mounted onto a patterned prelaminated PCB with four Au-sputtered pin headers (2.54 mm pitch), which served as terminals for four-point impedance measurement (Figure 1). The electrodes’ height was 1.2 mm and the coating thickness was 0.381 μm. SYLGARD™ 184 Silicone Elastomer Kit was used for filling a quarter of the glass tube with polydimethylsiloxane (PDMS); the polymer was cured for one hour in a convection oven set at 100 °C, resulting in a bottom-sealed well (h = 0.166 cm) with the Au-coated pins exposed, thus allowing contact with the nutrient fluid. Au showed no inhibitory effects on cell growth in in vitro cytotoxicity studies. The use of this type of electrode (vertical-positioned pins) contributed to direct interaction with the bulk solution and not only with the bottom layer, creating several shortcomings [36]. The bottom of the well was covered with a cured polydimethylsiloxane (PDMS) layer in order to obtain a cell-compatible environment. Due to its many advantages over other fabrication materials, PDMS received much attention as it constituted one of the most popular materials for the development of substrate platforms for mechanobiological [37,38] and microfluidic applications [39,40,41].

### 2.4. Electronic Interfacing

**Figure 2:**
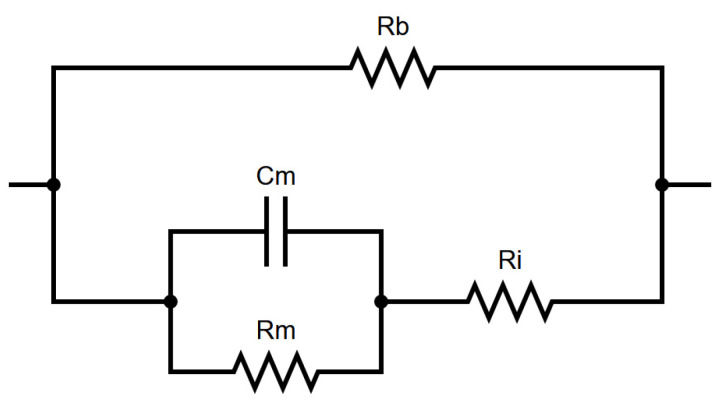
Equivalent electrical circuit for cell suspension in buffer.

An equivalent electrical circuit for measuring the cells’ impedance is presented in Figure 2. As seen from the equivalent circuit, the total measured impedance consisted of the buffer resistance (R_b_), the intracellular resistance (R_i_), and the cell membrane impedance, which included a resistor (R_m_) and a capacitor (C_m_) in parallel. The buffer resistance exhibited a purely ohmic behavior; the intracellular resistance was also modeled as a resistive junction (based on the extracellular fluids and proteins which were conductive) [42]. The cell impedance, in contrast to the other components, was frequency dependent, and so the applied frequencies should be chosen accordingly. The equivalent capacitor was based on the cell size; the larger the cell, the more lipid membrane was sandwiched between proteins, therefore increasing the capacitance. Based on the literature [11], the cell membrane resistance prevailed over the measurement due to the presence of the C_m_ element which acted as a bypass capacitor in a lower frequency range, impeding the current from passing through the intracellular space. Hence, the whole cell population in the buffer was treated as a unified resistor whose value depended on each cell membrane’s impedance. However, in a high-frequency range, the cell membrane impedance was considered as a high-pass filter and allowed the determination of the intracellular resistance, which had a much lower value compared to the total measured impedance. Therefore, by applying a frequency sweep, the cell contribution to the overall impedance could be highlighted, given the fact that the rest of the components of the system exhibited a purely ohmic, frequency-independent behavior.

**Figure 3:**
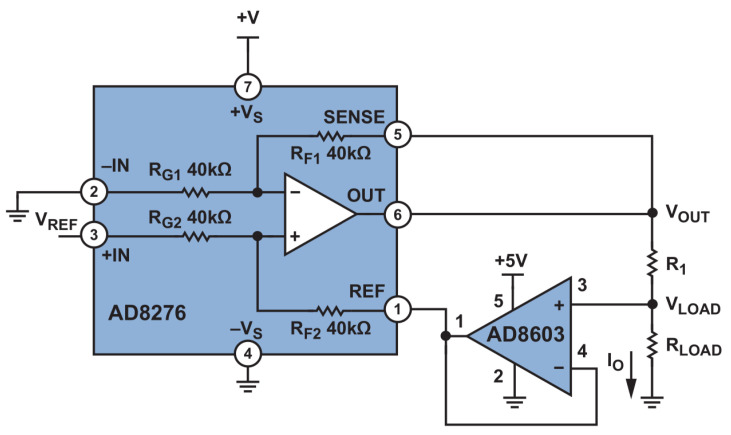
The Howland Pump circuit implemented using an AD8276 and a current buffer (AD8603), Adapted from Ref. [45].

**Figure 4:**
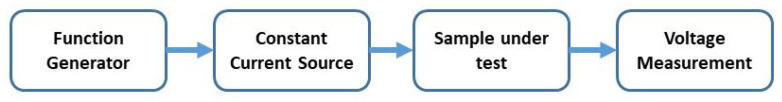
System block diagram.

For measuring the samples’ impedance at different frequencies, a Howland Pump circuit [43] (Figure 3) was implemented with a differential amplifier (AD8276) and a voltage buffer (AD8603). This circuit converted an input voltage (V_REF_) to an output current (I_O_) which was applied to a load (R_L_) without being affected by the load voltage drop. It was of great importance to the output impedance that the resistor ratios R_F1_/R_G1_ and R_F2_/R_G2_ were matched; for example, by using 1% tolerance 10 kΩ resistors, the worst-case output impedance was ±250 kΩ, which was unacceptable for high-precision applications [44]. Therefore, for the presented application an operational amplifier with highly matched trimmed built-in resistors was chosen. The proposed measurement system (Figure 4), which was an implementation of a Howland Pump circuit (Figure 3) was a voltage controlled current source and, by driving its input with varying frequency voltage waveforms generated by a Tektronix AFG3021 function generator, the resulting current waveforms were applied to the samples. This circuit topology was implemented in applications that required a steady current at a wide frequency bandwidth for bioimpedance measurements [45,46,47]. Output current was adjusted with V_REF_ and R_1_ applying a voltage to pin V_REF_ and selecting a resistor value (R_1_) (Figure 3). V_REF_ pin was a driver with the output of function generator, while in the setup high-precision resistors were used for R_1_. This design offered flexibility in adjusting the current applied to the samples simply by tuning the two aforementioned parameters; by driving the V_REF_ pin with a V_CC_ = ±2.5 V, R_1_ = 11.378 kΩ. A driving square wave of V_PP_ = 110 mV, an I_RMS_ = 10 μA was constantly provided to the samples via pins 1 and 4 (outer pins in Figure 1 which corresponded to pin 3 and ground of AD8603 in Figure 3), while voltage was measured using a Brymen BM867 handheld multimeter with TrueRMS in pins 2–4 (Figure 1). The setpoint current was selected to ensure that no damage occurred to the samples, while the generated voltage was detectable from the measurement equipment [11,41,48,49]. Additionally, by selecting an appropriate current, unwanted phenomena such as localized heating induced by current and excessive electrode polarization were avoided [50,51].

The four-point technique’s main advantage over the two-point and three-point impedance measurements was that by supplying a current from two points and by measuring the voltage using two other points, the effect of the electrode impedance, as well as the contact impedance, could practically be neglected. This configuration could be applied in order to obtain the precise values of the solution’s resistance separately or cell suspension [21].

## 3. Results-Discussion

In our study, the AC for impedance studies was applied for frequencies from 5 to 1000 Hz. Discrete frequency values of 5, 10, 100, 250, 500, and 1000 Hz were used for the evaluation. In each case, an increasing and a decreasing sweep was performed in the predefined frequency values and the average was calculated for each frequency. For every frequency, four cancer cell types (SK-N-SH, HeLa, MCF-7, and MDA-MB-231) were tested in two different population densities (50 K and 100 K cells/500 μL). For comparison purposes, DOX was applied to all cell lines in order to observe the impact of the anticancer agent as well as the alterations through the cell membrane’s reactions. Mean impedance values were calculated for each case of cell type/population, with and without the presence of DOX. The plain cell medium and the plain DOX in the plain cell culture medium were also tested for reference reasons.

**Figure 5:**
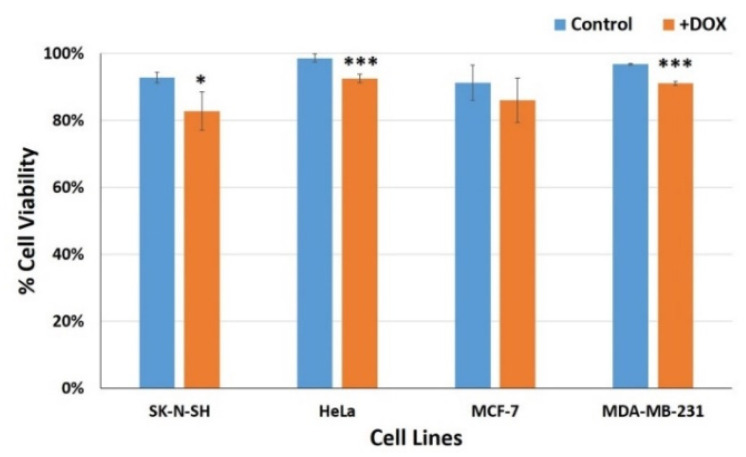
Graphic representation of the viability percentages of SK-N-SH, HeLa, MCF-7 and MDA-MB-231 cell lines before (blue columns) and after 30 min treatment with the anticancer agent doxorubicin (orange columns). * *p* < 0.05, *** *p* < 0.001 significantly different from the untreated control cells.

**Figure 6:**
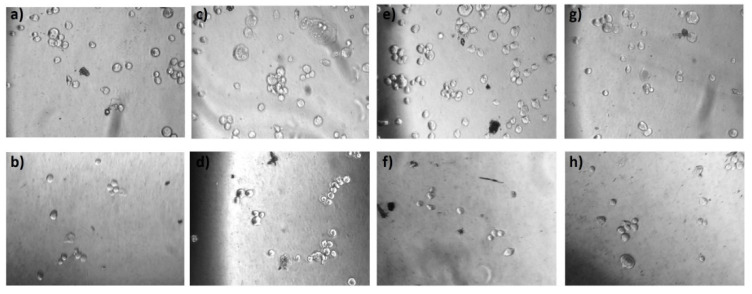
Morphological changes in the SK-N-SH (**a**,**b**), HeLa (**c**,**d**), MCF-7 (**e**,**f**) and MDA-MB-231 (**g**,**h**) cell lines before (first row) and after (second row) DOX treatment respectively. Scale bars = 50 μm.

In order to investigate the cell morphology throughout the time frame of a single measurement run, we examined the cells under a microscope. The 30 min incubation period was the average time of the experimental run in the frequency range chosen. At the first step, the four cancer cell lines were initially treated with 1 mM of DOX [4,52] for 30 min (the incubation time was based on the calculated duration of the impedance frequency sweeps) and cell viability percentages were counted (Figure 5). Our results were in accordance with a previous study conducted by Qiao et al. [11]. Even though there were no obvious changes in cell morphology (Figure 6), cervical HeLa, as well as the mammary MDA-MB-231 cell lines, presented the highest viability rates (98.7% and 96.8% respectively) that were slightly but significantly reduced after the DOX treatment at 92.5% and 91.2%, as predicted by previous studies [52]. Neuroblastoma SK-N-SH cells presented a lower initial viability (92.8%), whereas, in this case, DOX treatment caused a higher percentage of cell death (~10%). DOX exhibited no significant cytotoxic effects on the other breast cancer cell line, MCF-7, since they were considered a DOX-resistant cell model [53]. The DOX’s incubation time was chosen as the mean time of the experimental run and was considered a relatively short time, leading to ~10% alterations in the cells’ viability. An increase in the incubation period was expected to enhance the specific effect.

In the second step, the impedance evaluations were performed by the experimental setup that was described in previous sections.

**Figure 7:**
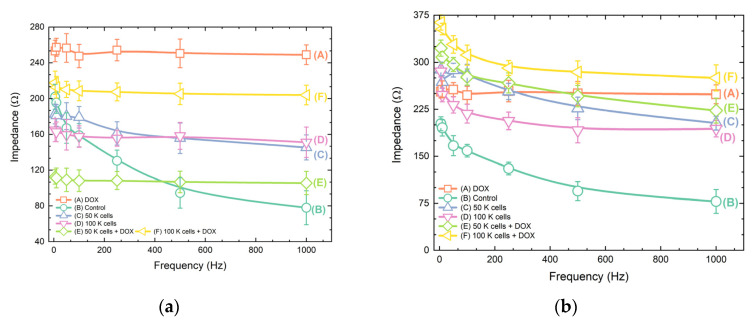

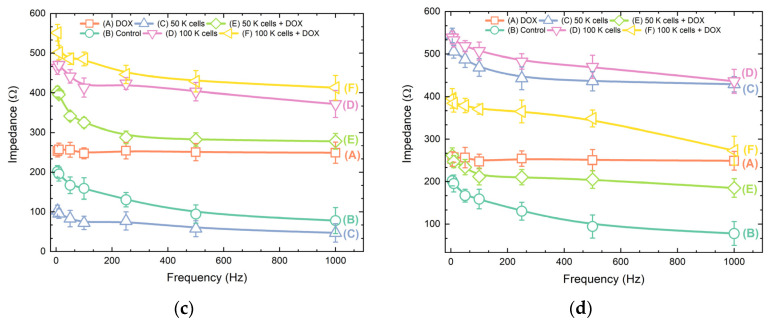
Mean impedance values of cell lines before and after treatment with the anticancer agent doxorubicin in two different population densities (50 K cells and 100 K cells/500 μL) in five different frequencies (5, 10, 100, 250, 500 and 1000 Hz): (**a**) SK-N-SH; (**b**) HeLa, (**c**) MCF-7; (**d**) MDA-MB-231.

Results indicate that for every cell line studied, the impedance decrease as frequency increases (Figure 7). In all graphs, the notation control and DOX represent the response of the medium solution and the response of the DOX solution, respectively. Both solutions do not include cells and are considered as a reference throughout the frequency range tested in every experimental case. In almost all cases, the impedance appears to be almost constant for frequencies above 100 Hz, indicating an ohmic behavior. In all the cases the impedance value tends to reach the same level for both population densities (50 K and 100 K cells/500 μL). An exception is observed in the MCF-7 (c) case where the impedance plateau is much higher for 100 K cells/500 μL. When DOX is applied to untreated cells a decrease in the impedance level is observed for the cases of SK-N-SH (50 K/500 μL) and MDA-MB-231 (50 K and 100 K/500 μL). In all the other cases an increase in the impedance is measured, indicating the reduction of the charged units into the solution. The absolute values are higher in the MCF-7 case (550 Ω) (c), whereas the SK-N-SH cell line (a) provides a lower impedance drop. We observe that the population of 50 K SK-N-SH cells/500 μL provides stronger signals in frequencies below 500 Hz than the respective 100 K cells/500 μL, but this behavior reverses when DOX is introduced to the system. An increase in impedance values relative to the cell population augmentation is observed in HeLa cells (b) treated with DOX, but the graph is completely mirrored in the absence of the anticancer agent. The population density of MCF-7 100 K cells/500 μL (c) gave the highest impedance values with DOX treatment, whereas MDA-MB-231 cells (d) demonstrated the maximum impedance responses at 100 K and 50 K cells/500 μL without the presence of DOX.

**Figure 8:**
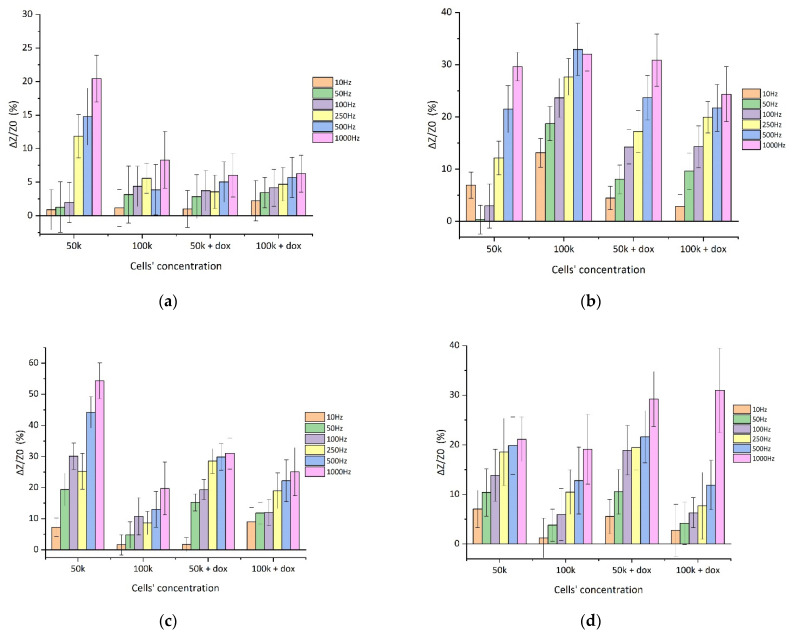
ΔZ/Z_0_ (%) for four cell lines in six different frequencies (10, 50, 100, 250, 500, and 1000 Hz) for two different population densities (50 K cells and 100 K cells/500 μL) before and after treatment with the anticancer agent doxorubicin: (**a**) SK-N-SH; (**b**) HeLa; (**c**) MCF-7; (**d**) MDA-MB-231.

Figure 8 illustrates the normalized impedance variations (ΔZ/Z_0_) as a function of the cells’ concentrations, including the standard values for several measurements in the same samples for all cell lines tested. The corresponding system sensitivity for each cell line can be extracted for the specific graphs. The ratio of the change in impedance is expressed as a function of frequency with the following Equation, by taking the absolute percentile value:

ΔZ/Z_0_ = |((Z − Z_0_ )∙Z_0_^−1^)∙100|, (2)

where Z_0_ was the impedance at the first frequency and Z is the alternating frequency impedance of the solution tested for every cell line, with or without DOX. As it is observed, the sensitivity is frequency-dependent whereas in all the cases it is dropping with the increase in the cells’ population. The limit of detection and resolution parameters are considered vital, nevertheless, no respective experiments have been carried out as of yet. Further ongoing research is being conducted toward this goal.

**Figure 9:**
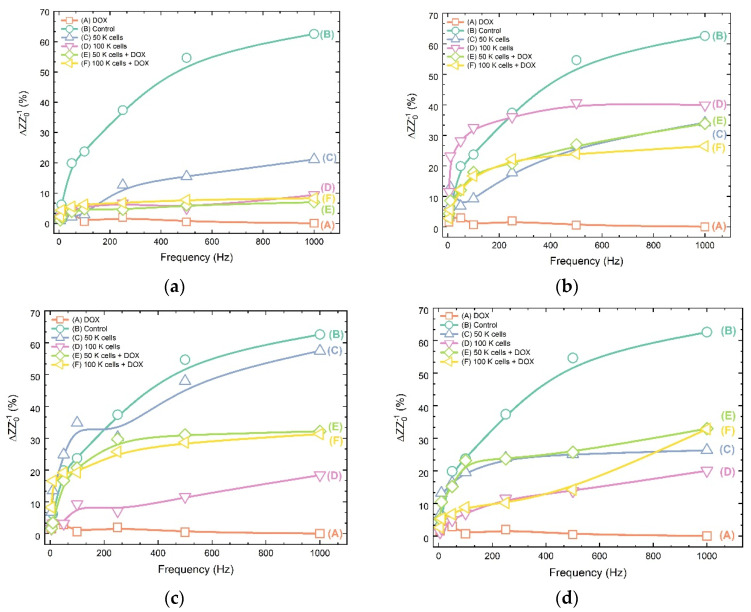
ΔZ/Z_0_ (%) for cell lines before and after treatment with the anticancer agent doxorubicin in two different population densities (50 K cells and 100 K cells/500 μL) in five different frequencies (5, 10, 100, 250, 500 and 1000 Hz): (**a**) SK-N-SH; (**b**) HeLa; (**c**) MCF-7; (**d**) MDA-MB-231.

The normalized impedance variations frequency curves of the four-point probe sensor under different cellular concentrations, treated or untreated with DOX, are shown in Figure 9. In all cases, the normalized impedance is higher at low frequencies (5–100 Hz) and drops at higher frequencies till a plateau is reached.

Generally, the differential impedance values for treated cells are very close for both population densities with the exception of case (c): MCF-7 (50 K cells). Furthermore, the high-frequency differential impedance values for the treated cells are close to 30 for all the cases, except for the SK-N-SH cells, where the corresponding values are in the area of 7. The distribution of cellular ΔZ/Z_0_ lies between the DOX (upper limit) and plain culture medium (lower limit) responses. Each cell line of different tissue origin presents a different profile. Thus, neuroblastoma SK-N-SH cell responses (a) are mainly gathered in the area up to 20, whereas the cervical HeLa cell line (b) provides ΔZ/Z_0_ dispersed between 20–40. Even in the case of the two breast cancer cell lines, MCF-7 (c) and MDA-MB-231 (d), we can observe variations in the responses. The estrogen-receptor positive MCF-7 cells provide ΔZ/Z_0_ values ranging between 5, whereas, in the case of the triple negative cell line MDA-MB-231, the values for the respective treatments vary within the area 20–30.

As an alternative to the four-electrode system, three-electrode systems for bioimpedance measurements are occasionally applied for low-frequency measurements of biological samples in order to counterbalance the polarization effect of the sensing electrode due to the formation of double-layer capacitance between the charged electrode surface and the available ions in the surrounding electrolytic solution [54,55]. In our study, we do not encounter this problem as demonstrated by the considerably low variability of ECIS measurements at each applied frequency. Four-electrode ECIS configurations are more commonly employed for the assessment of drug cancer-cell cytotoxic interactions [54]. In the same context, this could have been facilitated by the use of vertical, small-surface pin-shaped electrodes instead of planar ones, since it has been shown that impedance values are generally inversely proportional to the active working electrode area [56,57], with smaller-radius working electrodes generating more sensitive impedance shifts to cell density changes. The latter attribute was beyond the scope of our study, since impedance measurements were recorded soon after the application of the cytotoxic agent (DOX). For the same reason, we used pin-shaped electrodes (to cover as much possible of the vertical dimension of the cell suspension) instead of planar ones.

Further experimental sets will be needed in order to investigate the behavior of several cell suspensions from different tissue origins, as well as cell culture media and buffers of equivalent ionic strength. The importance of volume fraction will also need to be explored, using electrically conductive and electrically insulating materials (e.g., hydrogels and resins) in order to better understand the effect of the presence of cells in the total impedance value. The technique could be potentially used to predict cell bioelectric responses in tissue degeneration and biosensing applications, as well as an analytical tool to better understand ion kinetics in the presence of live tissues.

Bioelectric profiling of cancer cell responses to chemotherapeutic agents is an emerging research field, as indicated by another report [32] by our group of authors where impedance spectroscopy was applied in order to study interactions between the anticancer cytostatic agent 5-fluoruracil (5-FU) and four adherent mammalian cancer cells lines were immobilized in a three-dimensional (3D) calcium alginate hydrogel matrix, thus mimicking in vivo tissue conditions.

The transfer function for the equivalent electrical circuit of Figure 2 is extracted as:

(3) $$Z\left(\omega \right)=\left|\;\frac{j2{\pi \mathrm{fR}}_{b}R_{i}R_{m}C_{m}+R_{b}R_{i}+R_{m}R_{b}}{j2{\pi \mathrm{fR}}_{b}R_{m}C_{m}+j2{\pi \mathrm{fR}}_{i}R_{m}C_{m}+R_{b}+R_{i}+R_{m}}\;\right|\;,\;$$

where Ζ is the measured impedance, and the parameters R_b_, R_i_, R_m_ and C_m_ are the equivalent electrical components for each cell suspension. The fitting to the measured data presented in Figure 7 was performed with MATLAB (MathWorks Inc.), using the nonlinear least squares method. Results are presented in Table 1, Table 2, Table 3 and Table 4 alongside the goodness of fit.

According to the literature [11], the parameters C_m_ and R_m_ correspond to the membrane capacitance and resistance, respectively, while the parameter R_i_ reflects the intracellular resistance. From the above-mentioned tables, we can observe that the addition of the DOX induces a major impact in all the cell-related parameters, as expected. The resistance of the buffer which is represented by the parameter Rb does not seem to be related to the addition of the DOX, since the percentage of the solvent dimethylsulfoxide (DMSO) of DOX stock solution in the cell medium is negligible. Moreover, the parameter C_m_ for all the cases ranges between 10^−6^ and 10^−5^, indicating that the geometrical characteristics of the four cell lines under investigation do not exhibit major differences, which is the case in our evaluation.

This study demonstrates the ability of the bioimpedance technique to detect drug-cell interactions at low frequencies (up to 1 kHz). The identification of the non-observed effects of drug-cell interactions was also presented by Gelsinger et al. [58]. The authors stated that different frequencies could give higher resolution with the H1299 cell line during ECIS at the frequency range 250–1000 Hz for a 60-min time period. The main advantage of the utilization of low frequencies is that structural cell membrane alterations that could interfere with the instant effects of the cytotoxic agent could be avoided. This characteristic would facilitate the fingerprinting of drug effects without exposure to the cells for long time periods (e.g., for 24 or 48 h). On the same grounds, the proposed impedance system is supposed to be compatible with a cell suspension instead of adherent cell cultures.

## 4. Conclusions

The electrical impedance investigates the electrical behavior of cells which constitutes one of the main ways to understand cell function and provides valuable information about the dispersion caused by the cell membrane in a wide frequency range, depending on the membrane permittivity [59]. The electrical impedance cell-based biosensors are considered a beneficial approach based on the electrical signals, since they allow the direct analysis of the cellular activities that take place on the surface of an electrode and appear to be a critical tool for providing a quick, simple, and cheap technique to monitor numerous compounds for drug discovery at the very early stage [60,61,62,63].

Using the four-electrode configuration, the influence of the electrode polarization impedance can be reduced, compared to the two-electrode set-up where the electrode polarization impedance dominates during the measurement procedure, and thus the extraction of the contribution from the tissue with sufficient accuracy or sensitivity becomes difficult [29,64]. Measurements using a four-point probe setup can provide morphological information as they have a higher accuracy and sensitivity, especially in measuring the density of the biological tissues [21]. In addition, for bioimpedance measurements in a low-frequency range, the four-point probe is adopted for the determination of the impedance features [65].

In this work, highly sensitive four-point probe measurements were utilized in a frequency sweep sequence from 5 Hz to 1 KHz in order to extract differential cellular impedance responses from various cancer cell lines of different tissue origin. A custom-made electronic circuit was implemented for this purpose, based on the Howland Pump topology. Four cancer cell types were assessed, in two populations (50 K and 100 K cells) with and without treatment with DOX in order to study the impact of the anticancer agent as well as the alterations through the cell membrane’s reactions. A decrease in the impedance value was found with a frequency increase for all cases. Furthermore, the impedance value in high frequencies reaches almost the same level for both populations. When DOX is applied to a plain cell culture medium, a different behavior is observed for each cell type. All the measured values are fitted to the theoretical model and the main parameters of the impedance of the cell suspension are extracted in each case. The specific result indicates that a signature can potentially be extracted for each cell type in order to identify its response to the anticancer agent as a means of non-invasive cell status monitoring.

The present study is among the few using bioimpedance at a low frequency range (up to 1 kHz) to identify the previously unobserved effects of drug–cell interactions. As also shown by Gelsinger et al. for the H1299 cell line, a higher resolution between different frequencies can be achieved during ECIS at the frequency range 250–1000 Hz over an initial period of 60 min [58]. A low-frequency-based approach would help avoid structural cell membrane alterations that could interfere with the direct effect of the cytotoxic agent. This could be a vital performance trait of our method, since it enables the fingerprinting of drug effects without necessitating long periods (e.g., for 24 or 48 h) of cell culture treatment. For the same reason, the assay system is configured to be compatible with a cell suspension instead of attached cell cultures.

## Figures and Tables

**Table 1:** SK-N-SH-Electrical parameters of cell suspensions from the equivalent circuit.

	$C_{m}$	$R_{b}$	$R_{i}$	$R_{m}$	$R^{2}$
50 k	3.698 × 10^−6^	266.077	317.715	260.592	0.981
100 k	4.342 × 10^−5^	248.450	411.050	70.9458	0.773
50 k + DOX	3.240 × 10^−5^	139.636	454.263	109.0952	0.880
100 k + DOX	2.089 × 10^−5^	278.842	780.831	165.798	0.871

**Table 2:** HeLa-Electrical parameters of cell suspensions from the equivalent circuit.

	$C_{m}$	$R_{b}$	$R_{i}$	$R_{m}$	$R^{2}$
50 k	1.493 × 10^−6^	425.469	346.470	494.807	0.955
100 k	1.245 × 10^−5^	415.238	373.484	385.830	0.886
50 k + DOX	9.803 × 10^−6^	1309.046	283.377	124.010	0.912
100 k + DOX	6.640 × 10^−6^	531.136	596.051	503.853	0.980

**Table 3:** MCF-7-Electrical parameters of cell suspensions from the equivalent circuit.

	$C_{m}$	$R_{b}$	$R_{i}$	$R_{m}$	$R^{2}$
50 k	1.152 × 10^−5^	302.415	54.797	77.177	0.815
100 k	1.158 × 10^−5^	982.287	660.825	230.720	0.835
50 k + DOX	7.969 × 10^−6^	654.050	494.472	536.906	0.984
100 k + DOX	2.920 × 10^−6^	778.910	911.089	658.333	0.862

**Table 4:** MDA-ΜΒ-231-Electrical parameters of cell suspensions from the equivalent circuit.

	$C_{m}$	$R_{b}$	$R_{i}$	$R_{m}$	$R^{2}$
50 k	4.180 × 10^−6^	713.775	1106.618	885.778	0.925
100 k	2.854 × 10^−6^	1109.801	730.105	281.783	0.940
50 k + DOX	1.024 × 10^−5^	375.680	417.337	377.959	0.907
100 k + DOX	6.121 × 10^−6^	602.064	834.219	260.759	0.965

## Data Availability

The data presented in this study are available upon request.

## References

[1] Fitzmaurice C., Dicker D., Pain A., Hamavid H., Moradi-Lakeh M., MacIntyre M.F., Allen C., Hansen G., Woodbrook R., Wolfe C. (2015). The Global Burden of Cancer 2013. JAMA Oncol..

[2] Han K.-H., Han A., Frazier A.B. (2006). Microsystems for isolation and electrophysiological analysis of breast cancer cells from blood. Biosens. Bioelectron..

[3] Dubbelboer I.R., Pavlovic N., Heindryckx F., Sjögren E., Lennernäs H. (2019). Liver Cancer Cell Lines Treated with Doxorubicin under Normoxia and Hypoxia: Cell Viability and Oncologic Protein Profile. Cancers.

[4] Minotti G., Menna P., Salvatorelli E., Cairo G., Gianni L. (2004). Anthracyclines: Molecular advances and pharmacologic developments in antitumor activity and cardiotoxicity. Pharmacol. Rev..

[5] Tacar O., Sriamornsak P., Dass C.R. (2013). Doxorubicin: An update on anticancer molecular action, toxicity and novel drug delivery systems. J. Pharm. Pharmacol..

[6] Ackmann J.J. (1993). Complex bioelectric impedance measurement system for the frequency range from 5 Hz to 1 MHz. Ann. Biomed. Eng..

[7] Ackmann J.J., Seitz M.A. (1984). Methods of complex impedance measurements in biologic tissue. Crit. Rev. Biomed. Eng..

[8] Cha K., Chertow G.M., Gonzalez J., Lazarus J.M., Wilmore D.W. (1995). Multifrequency bioelectrical impedance estimates the distribution of body water. J. Appl. Physiol..

[9] Bera T.K., Nagaraju J. (2011). Electrical Impedance Spectroscopic Studies on Broiler Chicken Tissue Suitable for the Development of Practical Phantoms in Multifrequency EIT. J. Electr. Bioimpedance.

[10] Bauchot A.D., Harker F.R., Arnold W.M. (2000). The use of electrical impedance spectroscopy to assess the physiological condition of kiwifruit. Postharvest Biol. Technol..

[11] Qiao G., Wang W., Duan W., Zheng F., Sinclair A.J., Chatwin C.R. (2012). Bioimpedance Analysis for the Characterization of Breast Cancer Cells in Suspension. IEEE Trans. Biomed. Eng..

[12] Grimnes S., Martinsen O. (2014). Bioimpedance & Bioelectricity Basics.

[13] Ruiz-Vargas A., Ivorra A., Arkwright J.W. (2018). Design, Construction and Validation of an Electrical Impedance Probe with Contact Force and Temperature Sensors Suitable for in-vivo Measurements. Sci. Rep..

[14] Two, Three and Four Electrode Experiments—The Number of Electrodes (or Probes) Used Two, Three, Four Electrode. https://www.gamry.com/application-notes/instrumentation/two-three-four-electrode-experiments/.

[15] Waremra R.S., Betaubun P. (2018). Analysis of Electrical Properties Using the four point Probe Method. E3S Web Conf..

[16] Schuetze A.P., Lewis W., Brown C., Geerts W.J. (2004). A laboratory on the four-point probe technique. Am. J. Phys..

[17] Geddes L.A., Roeder R. (2003). Criteria for the Selection of Materials for Implanted Electrodes. Ann. Biomed. Eng..

[18] Yúfera A., Rueda A. A Method for Bioimpedance Measure With Four- and Two-Electrode Sensor Systems. Proceedings of the 30th Annual International IEEE EMBS Conference.

[19] Huang X. (2004). Simulation of Microelectrode Impedance Changes Due to Cell Growth. IEEE Sens. J..

[20] Holder D. (2005). Electrical Impedance Tomography: Methods, History and Applications.

[21] Amini M., Hisdal J., Kalvøy H. (2018). Applications of bioimpedance measurement techniques in tissue engineering. J. Electr. Bioimpedance.

[22] Crowell L.L., Yakisich J.S., Aufderheide B., Adams T.N.G. (2020). Electrical Impedance Spectroscopy for Monitoring Chemoresistance of Cancer Cells. Micromachines.

[23] Morgan H., Sun T., Holmes D., Gawad S., Green N.G. (2006). Single cell dielectric spectroscopy. J. Phys. D Appl. Phys..

[24] Lei K.F. (2014). Review on Impedance Detection of Cellular Responses in Micro/Nano Environment. Micromachines.

[25] Hassan Q., Ahmadi S., Kerman K. (2020). Recent Advances in Monitoring Cell Behavior Using Cell-Based Impedance Spectroscopy. Micromachines.

[26] Pan Y., Hu N., Wei X., Gong L., Zhang B., Wan H., Wang P. (2019). 3D cell-based biosensor for cell viability and drug assessment by 3D electric cell/matrigel-substrate impedance sensing. Biosens. Bioelectron..

[27] Seidel D., Rothe R., Kirsten M., Jahnke H.G., Dumann K., Ziemer M., Robitzki A.A. (2019). A multidimensional impedance platform for the real-time analysis of single and combination drug pharmacology in patient-derived viable melanoma models. Biosens. Bioelectron..

[28] Bragós R., Sarró E., Estruch H., Farré J., Cairó J., Bayés-Genís A., Gòdia F. Cell Growing and Differentiation Monitoring System using Electrical Bioimpedance Spectroscopy Measurement on Interdigitated Microelectrodes. Proceedings of the 3rd European Medical and Biological Engineering Conference November.

[29] Bragos R., Sarro E., Fontova A., Soley A., Cairó J., Bayes-Genis A., Rosell J. Four versus two-electrode measurement strategies for cell growing and differentiation monitoring using electrical impedance spectroscopy. Proceedings of the Annual International Conference of the IEEE Engineering in Medicine & Biology Society.

[30] Wu H., Zhou W., Yang Y., Jia J., Bagnaninchi P. (2018). Exploring the Potential of Electrical Impedance Tomography for Tissue Engineering Applications. Materials.

[31] Yang L., Arias L.R., Lane T.S., Yancey M.D., Mamouni J. (2011). Real-time electrical impedance-based measurement to distinguish oral cancer cells and non-cancer oral epithelial cells. Anal. Bioanal. Chem..

[32] Paivana G., Mavrikou S., Kaltsas G., Kintzios S. (2019). Bioelectrical Analysis of Various Cancer Cell Types Immobilized in 3D Matrix and Cultured in 3D-Printed Well. Biosensors.

[33] McKenna M.T., Weis J.A., Barnes S.L., Tyson D.R., Miga M.I., Quaranta V., Yankeelov T.E. (2017). A Predictive Mathematical Modeling Approach for the Study of Doxorubicin Treatment in Triple Negative Breast Cancer. Sci. Rep..

[34] Shapiro H.M. (1988). Practical Flow Cytometry.

[35] Li J., Wang Y., Ba D. (2012). Characterization of Semiconductor Surface Conductivity by Using Microscopic Four-Point Probe Technique. Phys. Procedia.

[36] Selvakumaran J., Hughes M.P., Keddie J.L., Ewins D.J. Assessing biocompatibility of materials for implantable microelectrodes using cytotoxicity and protein adsorption studies. Proceedings of the 2nd Annual International IEEE-EMBS Special Topic Conference on Microtechnologies in Medicine and Biology.

[37] Brown X.Q., Ookawa K., Wong J.Y. (2005). Evaluation of polydimethylsiloxane scaffolds with physiologically-relevant elastic moduli: Interplay of substrate mechanics and surface chemistry effects on vascular smooth muscle cell response. Biomaterials..

[38] Park J.Y., Yoo S.J., Lee E.J., Lee D.H., Kim J.Y., Lee S.H. (2010). Increased poly(dimethylsiloxane) stiffness improves viability and morphology of mouse fibroblast cells. BioChip J..

[39] Chung S., Sudo R., Mack P.J., Wan C.R., Vickerman V., Kamm R.D. (2009). Cell migration into scaffolds under co-culture conditions in a microfluidic platform. Lab Chip.

[40] Huh D., Hamilton G.A., Ingber D.E. (2011). From 3D cell culture to organs-on-chips. Trends Cell Biol..

[41] Menon N.V., Chuah Y.J., Cao B., Lim M., Kang Y. (2014). A microfluidic co-culture system to monitor tumor-stromal interactions on a chip. Biomicrofluidics.

[42] Kadan-Jamal K., Sophocleous M., Jog A., Desagani D., Teig-Sussholz O., Georgiou J., Shacham-Diamand Y. (2020). Electrical Impedance Spectroscopy of plant cells in aqueous biological buffer solutions and their modelling using a unified electrical equivalent circuit over a wide frequency range: 4Hz to 20 GHz. Biosens. Bioelectron..

[43] Mahnam A., Yazdanian H., Samani M.M. (2016). Comprehensive study of Howland circuit with non-ideal components to design high performance current pumps. Measurement.

[44] Pease R.A. (2008). A comprehensive study of the Howland current pump—AN-1515. Natl. Semiconductor.

[45] Bertemes-Filho P., Vincence V.C., Santos M.M., Zanatta I.X. (2012). Low power current sources for bioimpedance measurements: A comparison between Howland and OTA-based CMOS circuits. J. Electr. Bioimpedance.

[46] Han B., Xu Y., Dong F. (2017). Design of current source for multi-frequency simultaneous electrical impedance tomography. Rev. Sci. Instrum..

[47] Liu J., Qiao X., Wang M., Zhang W., Li G., Lin L. (2014). The differential Howland current source with high signal to noise ratio for bioimpedance measurement system. Rev. Sci. Instrum..

[48] Ibrahim B., Jafari R. (2019). Cuffless Blood Pressure Monitoring from an Array of Wrist Bio-impedance Sensors using Subject-Specific Regression Models: Proof of Concept. IEEE Trans. Biomed. Circuits Syst..

[49] Hinrichs P., Cagle J.C., Sanders J.E. (2019). A portable bioimpedance instrument for monitoring residual limb fluid volume in people with transtibial limb loss: A technical note. Med. Eng. Phys..

[50] Asphahani F., Wang K., Thein M., Veiseh O., Yung S., Xu J., Zhang M. (2011). Single-cell bioelectrical impedance platform for monitoring cellular response to drug treatment. Phys. Biol..

[51] Griffiths H., Tucker M.G., Sage J., Herrenden-Harker W.G. (1996). An electrical impedance tomography microscope. Physiol. Meas..

[52] Kibria G., Hatakeyama H., Akiyama K., Hida K., Harashima H. (2014). Comparative Study of the Sensitivities of Cancer Cells to Doxorubicin, and Relationships between the Effect of the Drug-Efflux Pump P-gp. Biol. Pharm. Bull..

[53] Lovitt C.J., Shelper T.B., Avery V.M. (2018). Doxorubicin resistance in breast cancer cells is mediated by extracellular matrix proteins. BMC Cancer.

[54] Pradhan R., Kalkal A., Jindal S., Packirisamy G., Manhas S. (2021). Four electrode-based impedimetric biosensors for evaluating cytotoxicity of tamoxifen on cervical cancer cells. RSC Adv..

[55] Schwan H.P. (1966). Alternating current electrode polarization. Radiat. Environ. Biophys..

[56] Pradhan R., Mitra A., Das S. (2012). Impedimetric characterization of human blood using three-electrode based ECIS devices. J. Electr. Bioimpedance.

[57] Zhang X., Wang W., Nordin A.N., Li F., Jang S., Voiculescu I. (2017). The influence of the electrode dimension on the detection sensitivity of electric cell–substrate impedance sensing (ECIS) and its mathematical modeling. Sens. Actuators B Chem..

[58] Gelsinger M.L., Tupper L.L., Matteson D.S. (2019). Cell Line Classification Using Electric Cell-Substrate Impedance Sensing (ECIS). Int. J. Biostat..

[59] Le H., Kim J., Park J., Cho S. (2019). A Review of Electrical Impedance Characterization of Cells for Label-Free and Real-Time Assays. BioChip J..

[60] Asphahani F., Zhang M. (2007). Cellular impedance biosensors for drug screening and toxin detection. Analyst.

[61] Halai R., Cooper M.A. (2006). Current biosensor technologies in drug discovery. Drug Discov. World.

[62] Liu Q., Wu C., Cai H., Hu N., Zhou J., Wang P. (2014). Cell-Based Biosensors and Their Application in Biomedicine. Chem. Rev..

[63] Huerta-Nuñez L.F.E., Gutierrez-Iglesias G., Martínez-Cuazitl A., Mata-Miranda M.M., Alvarez-Jiménez V.D., Sánchez-Monroy V., Golberg A., González-Díaz C.A. (2019). A biosensor capable of identifying low quantities of breast cancer cells by electrical impedance spectroscopy. Sci. Rep..

[64] Sarro E., Fontova A., Soley A., Cairo J., Bayes-Genis A., Rosell J., Bragós R. (2007). Four electrode EIS measurement on interdigitated microelectrodes for adherent cell growing and differentiation monitoring. Proceedings of the 13th International Conference on Electrical Bioimpedance and the 8th Conference on Electrical Impedance Tomography—IFMBE, Prague, Czech Republic, 20–25 November 2005.

[65] Ivorra Cano A. (2004). Contributions to the Measurement of Electrical Impedance for Living Tissue Ischemia Injury Monitoring.

